# Revealing the Lattice Carbonate Mediated Mechanism in Cu_2_(OH)_2_CO_3_ for Electrocatalytic Reduction of CO_2_ to C_2_H_4_

**DOI:** 10.1002/advs.202308949

**Published:** 2024-02-04

**Authors:** Yugang Gao, Difei Xiao, Zeyan Wang, Zhaoke Zheng, Peng Wang, Hefeng Cheng, Yuanyuan Liu, Ying Dai, Baibiao Huang

**Affiliations:** ^1^ State Key Laboratory of Crystal Materials Shandong University Jinan 250100 China; ^2^ School of Physics Shandong University Jinan 250100 China

**Keywords:** Cu_2_(OH)_2_CO_3_, electrocatalytic CO_2_ reduction, ethylene, lattice carbonate mediated mechanism, selectivity

## Abstract

Understanding the CO_2_ transformation mechanism on materials is essential for the design of efficient electrocatalysts for CO_2_ reduction. In aconventional adsorbate evolution mechanism (AEM), the catalysts encounter multiple high‐energy barrier steps, especially CO_2_ activation, limiting the activity and selectivity. Here, lattice carbonate from Cu_2_(OH)_2_CO_3_ is revealed to be a mediator between CO_2_ molecules and catalyst during CO_2_ electroreduction by a ^13^C isotope labeling method, which can bypass the high energy barrier of CO_2_ activation and strongly enhance the performance. With the lattice carbonate mediated mechanism (LCMM), the Cu_2_(OH)_2_CO_3_ electrode exhibited ten‐fold faradaic efficiency and 15‐fold current density for ethylene production than the Cu_2_O electrode with AEM at a low overpotential. Theoretical calculations and in situ Raman spectroscopy results show that symmetric vibration of carbonate is precisely enhanced on the catalyst surface with LCMM, leading to faster electron transfer, and lower energy barriers of CO_2_ activation and carbon–carbon coupling. This work provides a route to develop efficient electrocatalysts for CO_2_ reduction based on lattice‐mediated mechanism.

## Graphical abstract

Lattice carbonate in Cu_2_(OH)_2_CO_3_ is found to be a mediator linking CO_2_ molecules with catalyst during CO_2_ electroreduction by a ^13^C isotope labeling experiment. Following the lattice carbonate mediated mechanism (LCMM), the Cu_2_(OH)_2_CO_3_ electrode exhibits enhanced ethylene selectivity and activity, which is attributed to the fast electron transfer and reduced energy barriers of CO_2_ activation and carbon–carbon coupling.

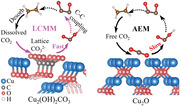

## 1 Introduction

Electrocatalytic CO_2_ reduction reaction (CO_2_RR) is regarded as one of the most promising strategies to mitigate energy and environmental problems concerning CO_2_ emission,^[^ ^1^ ^]^ which can convert CO_2_ and H_2_O into fuels or chemicals under mild reaction conditions with controllable reaction rates and product selectivity.^[^ ^2^ ^]^ Great efforts have been made in the past few decades with the purpose to explore efficient catalysts that can convert CO_2_ into chemicals with high activity and selectivity.^[^ ^3^ ^]^ However, the performance of the catalysts, especially for CO_2_ to multi‐carbon (C_2+_) products, is still unsatisfactory owing to the slow multi‐electron transfer process and controversial reaction pathways.^[^ ^4^ ^]^

As the activation of CO_2_ and the C─C coupling were regarded as the key steps for electrocatalytic CO_2_RR to C_2+_ transformation, most of the present catalysts were developed by optimizing the adsorption^[^ ^5^ ^]^ of CO_2_ on the surface of the catalysts and mediating the stability of the intermediates to promote the C─C coupling.^[^ ^6^ ^]^ However, in most of the cases, the participation of the atoms from the catalysts was not considered. As widely demonstrated in many other electrochemical processes, such as aromatic hydrocarbon oxidation,^[^ ^7^ ^]^ oxygen evolution reaction (OER),^[^ ^8^ ^]^ nitrogen reduction reaction (NRR),^[^ ^9^ ^]^ and anionic redox reaction in lithium‐ion batteries,^[^ ^10^ ^]^ etc., the participation of the atoms from the crystal lattice of the catalysts could not only be advantageous for the activation of the reactants by lowering the energy barriers, but also boost the reaction kinetics. For example, during the OER process in alkali conditions, the lattice oxygen atoms from the catalysts were found to be able to couple with hydroxide directly, instead of the coupling between hydroxide groups under conventional adsorbate evolution mechanism (AEM), which results in the improvement on the intrinsic OER activity.^[^ ^11^ ^]^ This reminds us to consider whether the lattice‐involved mechanism could also exist in the CO_2_RR process, and affect the electrocatalytic CO_2_RR to C_2+_ performances, which could be a new route to fabricate efficient electrocatalysts for CO_2_RR.

According to the literatures, the electrocatalysts with lattice‐involved mechanism usually contain the same atoms as in the reactants, i.e., the catalysts for OER and NRR typically contain large amounts of O and N atoms, respectively, the electrocatalysts, in which the atoms may participate the CO_2_RR process, should contain C atoms. Regarding the possible carbon‐containing species, carbonates are being found to be reactive intermediates during CO_2_RR due to their equilibrium with bicarbonate.^[^ ^12^ ^]^ Moreover, carbonate intermediates in situ generated on catalyst surface during CO_2_RR have been found to promote CO_2_RR kinetics, both in photocatalysis^[^ ^13^ ^]^ and electrocatalysis.^[^ ^14^ ^]^ Further, recently, Ma et al. reported a direct carbonate electroreduction to formate.^[^ ^15^ ^]^ Based on these considerations, Cu_2_(OH)_2_CO_3_, containing carbonate groups and Cu that is widely used in C_2_ production, was chosen as the candidate to investigate whether the lattice carbonate could be involved in the CO_2_RR to C_2_ products. Combined ^13^C stable isotope ratio mass spectrometry (IRMS) tests with experiments, we proved that lattice carbonate could act as a mediator to connect external CO_2_ molecules to active Cu atoms on Cu_2_(OH)_2_CO_3_ surface, which is named lattice carbonate mediated mechanism (LCMM). Density functional theory (DFT) calculations and in situ Raman spectrometry is performed to show that the symmetric vibration of carbonates on LCMM catalyst is greatly enhanced, thereby promoting CO_2_ activation and carbon–carbon coupling in C_2_H_4_ production. With LCMM, the as‐prepared Cu_2_(OH)_2_CO_3_ electrode exhibited the highest C_2_H_4_ faradaic efficiency (FE_C2H4_) of 60% at −0.9 V relative to RHE (see Figure S1, Supporting Information for RHE calibration), far better than that of 28% at −1.2 V for Cu_2_O electrode with conventional AEM.

## 2 Results and Discussion

**Figure 1:**
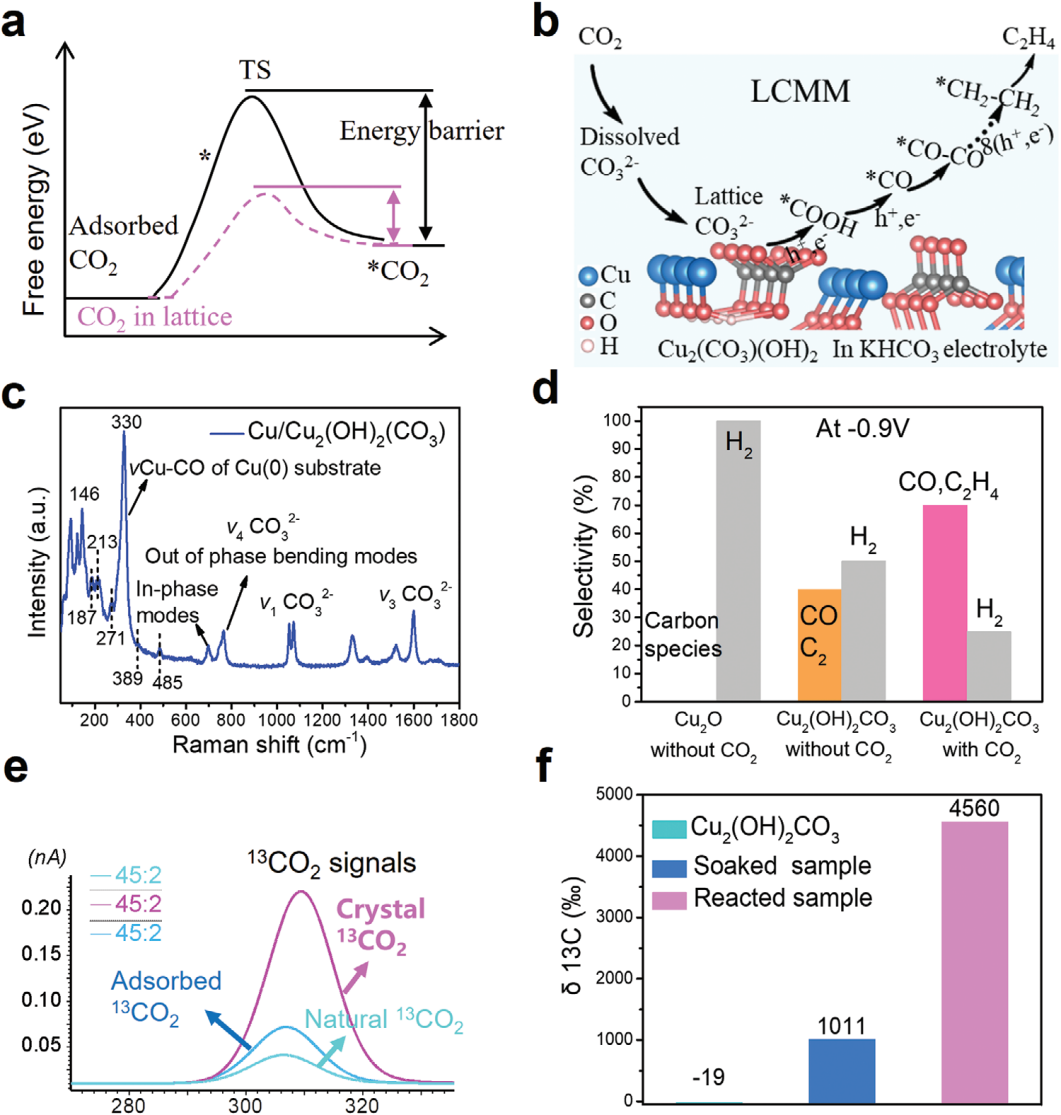
a) Free energies schematics of CO_2_ activation for conventional process and lattice involved process, b) Proposed lattice carbonate mediated mechanism (LCMM) in Cu_2_(OH)_2_CO_3_, c) Raman spectra for the as‐prepared sample, d) Comparison of the products from Cu_2_O and Cu_2_(OH)_2_CO_3_ electrodes without CO_2_, e) ^13^C intensities f) δ ^13^C values for unreacted, soaked and reacted Cu_2_(OH)_2_CO_3_ electrodes calculated by IRMS.

Generally, CO_2_ activation is very difficult during CO_2_RR, but if the lattice carbonate in the catalyst can be used as a mediator to perform the functions of CO_2_ adsorption and conversion, the activation energy barrier may be reduced (**Figure** **1**a). The schematic process of LCMM is shown in Figure 1b, indicating that lattice carbonate‐mediated CO_2_RR includes two key steps, namely the direct reduction of lattice carbonate and the regeneration of lattice carbonate after it has captured surrounding CO_2_. To prove these two steps, we grew a layer of Cu_2_(OH)_2_CO_3_ nanoparticles on Cu foil by an electro‐oxidation method (for details, see the experimental section of supporting information, SI) for validation experiments. Raman spectra were shown in Figure 1c, indicating that most of the vibration peaks in the fingerprint region, such as 146, 187, 271, 764, 1053, and 1600 cm^−2^ etc., belong to Cu_2_(OH)_2_CO_3_.^[^ ^14^, ^16^ ^]^ First, the potentiostatic measurements for Cu_2_(OH)_2_CO_3_ and Cu_2_O electrodes were performed in an H‐cell with a CO_2_‐free Na_2_SO_4_ electrolyte at −0.9 V and the products were then determined by gas chromatography (GC) (Figure S2, Supporting Information). We detected CO_2_ reduction products (CO and C_2_H_6_) from the Cu_2_(OH)_2_CO_3_ electrode instead of the Cu_2_O electrode (Figure 1d), indicating that lattice carbonate can be reduced not only to CO,^[^ ^14b^ ^]^ but also to C_2_ products. Second, after lattice carbonate is reduced, whether it can be regenerated by capturing the CO_2_ in the electrolyte is also important. We envisioned that if the ^13^CO_2_ could be transferred into the lattice of the electrocatalyst during the reaction, ^13^C signal in the Cu_2_(OH)_2_CO_3_ electrode after the reaction should be enhanced. Following this idea, we conducted potentiostatic measurements for the same electrode at −0.9 V, but in a ^13^CO_2_‐saturated KHCO_3_ electrolyte. We then used ^13^C IRMS (for details, see the experimental section of SI) to evaluate the amount of ^13^C in Cu_2_(OH)_2_CO_3_. After electrocatalytic ^13^CO_2_RR, the ^13^C intensity (Figure 1e) and δ ^13^C value (Figure 1f) of Cu_2_(OH)_2_CO_3_ measured by IRMS were both greatly enhanced compared with the other samples without reaction, indicating that ^13^CO_2_ could be transferred into crystal lattice of Cu_2_(OH)_2_CO_3_ during the reaction. Combining the above results, we experimentally prove that LCMM can be implemented in electrocatalytic CO_2_RR. Furthermore, gas chromatography mass spectrometry (GCMS) was also used to detect the gases after the reaction, showing that ^13^C_2_H_4_ and ^13^CO were the main products. The intensity of ^13^C_2_H_4_ was much higher than that of ^13^CO (Figure S3a, Supporting Information). The corresponding mass chromatograph also confirmed the above results (Figure S3b, Supporting Information). Thus, ^13^CO_2_ molecules are ultimately electrochemically reduced to value‐added products, especially ^13^C_2_H_4_, through LCMM in Cu_2_(OH)_2_CO_3_.

**Figure 2:**
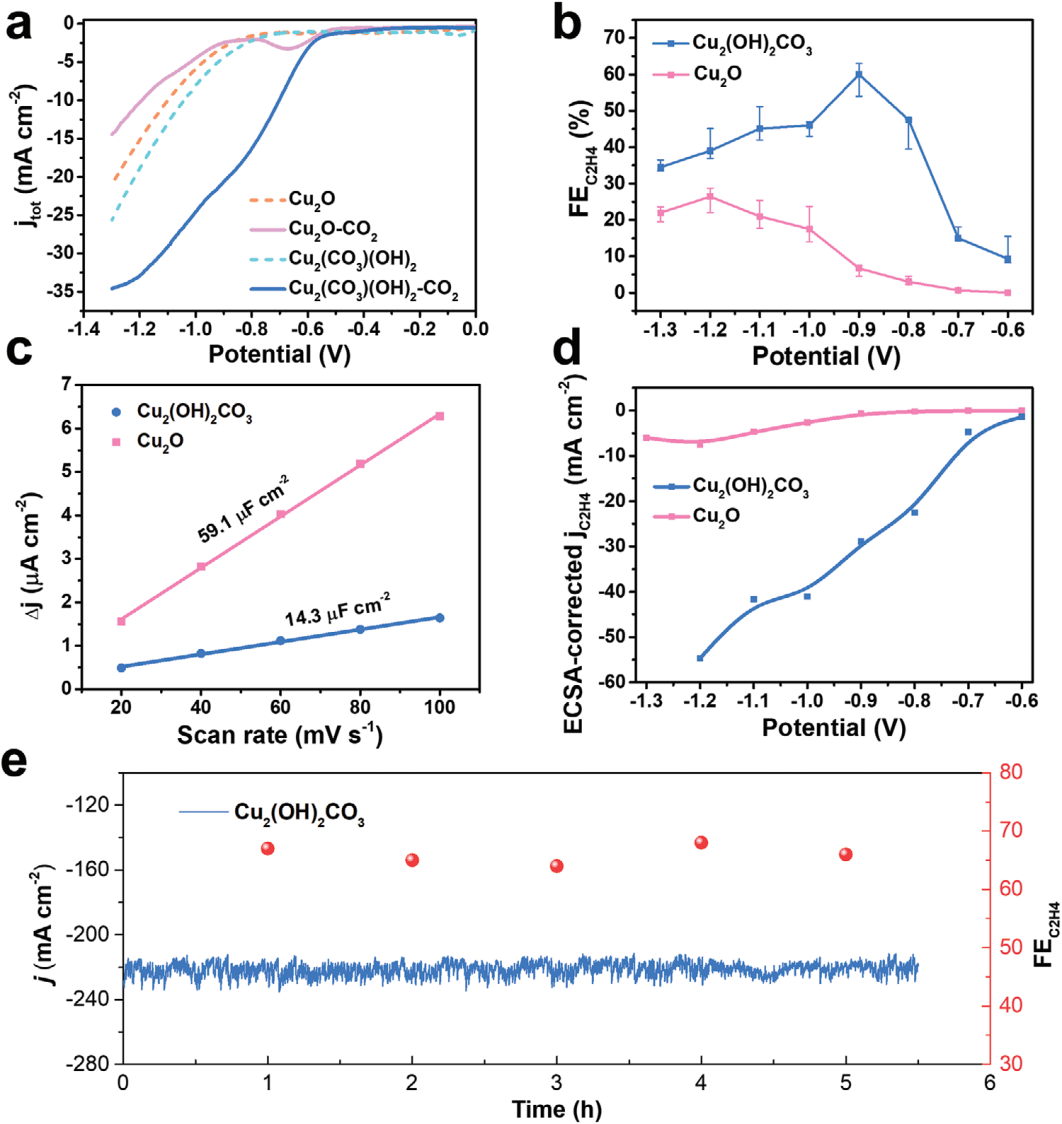
a) LSV curves, b) C_2_H_4_ faradaic efficiencies, c) Specific capacitances curves at various scan rates for calculating ECSA, d) Intrinsic current densities corrected by ECSA in H‐cells, e) FE_C2H4_ and current density values over time in flow cells.

To determine how LCMM affected the electrocatalytic CO_2_ conversion to C_2+_ products, two electrodes, namely Cu_2_(OH)_2_CO_3_ electrode with LCMM and Cu_2_O electrode with conventional AEM were prepared by depositing their nanoparticles on glassy carbon electrode (GCE), respectively. Their crystal structure and electronic structure were confirmed by various characterizations (Figures S4 and S5, Supporting Information). Linear sweep voltammetry (LSV) measurements were carried out for these two electrodes at a 20 mV s^−1^ scan rate. As seen in **Figure** **2**a, the total current densities, j_tot_, of Cu_2_(OH)_2_CO_3_ electrode increase significantly after the bubbling of CO_2_, while that of Cu_2_O electrode increase gently under the same conditions, indicating a higher catalytic activity of Cu_2_(OH)_2_CO_3_. To evaluate the selectivity of these electrodes, their FE_C2H4_ values in 3 h were shown in Figure 2b, presenting that FE_C2H4_ values of Cu_2_(OH)_2_CO_3_ electrode are higher than that of Cu_2_O electrode at a wide range of potentials from −0.6 to −1.3 V. The highest FE_C2H4_ value for Cu_2_(OH)_2_CO_3_ was ≈60% at −0.9 V, which was ≈1 order and 2 orders of magnitude higher than that of Cu_2_O (FE_C2H4_≈6%) and Cu (FE_C2H4_≈0.5%, Figure S6a, Supporting Information), respectively. Then, we further evaluated their intrinsic C_2_H_4_ current densities, j_C2H4_, which were j_C2H4_ values (Figure S6b, Supporting Information) corrected by electrochemical surface area (ECSA). The ECSAs were calculated by determining their specific capacitances (Figure 2c) and roughness factor (*R*_f_) from cyclic voltammetry measurements^[^ ^17^ ^]^ (Figure S7, Supporting Information). As summarized in Figure 2d, intrinsic j_C2H4_ value of the Cu_2_(OH)_2_CO_3_ electrode was ≈29.0 mA cm^−2^, which is 41‐fold higher of that of Cu_2_O (≈0.70 mA cm^−2^) at −0.9 V. Notably, the selectivity and activity of Cu_2_(OH)_2_CO_3_ electrode at a low overpotential are better that those of most reported catalysts under similar conditions as seen in Table S1 (Supporting Information). We then applied the catalyst in Flow cells (Figure 2e) and obtained an industrial current density of ≈220 mA cm^−2^ and FE_C2H4_ of ∼65% in 5 h, presenting great value in practical applications. Electrochemical impedance spectra^[^ ^18^ ^]^ (EIS) of these two electrodes recorded at −0.6 V were also shown in Figure S8 (Supporting Information), indicating that the electron transfer in the interface of catalyst and electrolyte is also easier for Cu_2_(OH)_2_CO_3_ electrode. In short, the Cu_2_(OH)_2_CO_3_ electrode with LCMM has faster catalytic reaction kinetic of CO_2_ conversion to C_2_H_4_, namely, higher C_2_H_4_ selectivity and activity at lower overpotential than that with AEM.

**Figure 3:**
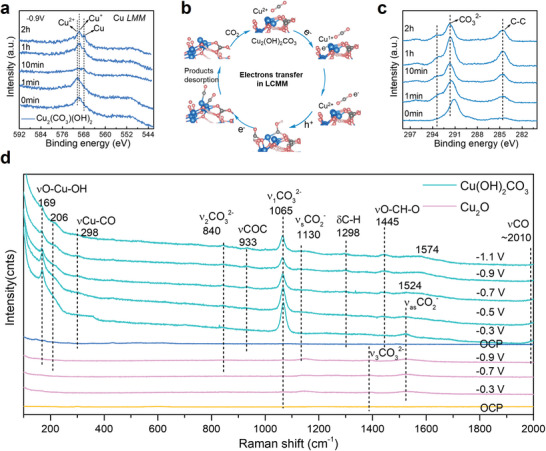
Structure and reaction intermediates evolution. a) Cu *LMM* Auger spectra for Cu_2_(OH)_2_CO_3_ electrode at different reaction times, b) Possible electron transfer path in LCMM, c) High‐resolution XPS spectra of C1s for Cu_2_(OH)_2_CO_3_ electrode at different reaction times, d) In situ Raman spectra for Cu_2_(OH)_2_CO_3_ and Cu_2_O electrodes at several potentials.

To better understand this observation, we clarify the detailed reaction process by characterizing the evolution of structure and surface intermediates for these electrodes. **Figure** **3**a presents the Cu *LMM* Auger spectra of Cu_2_(OH)_2_CO_3_^[^ ^19^ ^]^ at different reaction times, showing that Cu^2+^ in Cu_2_(OH)_2_CO_3_ is first partially reduced to Cu^1+^ and Cu^0^ in 10 min and then regenerated after 1 h. This result is also supported by High‐resolution TEM images with the lattice fringes of Cu_2_(OH)_2_CO_3_ after reaction (Figures S9 and S10, Supporting Information). This valence states changes of Cu atoms may reflect the electron transfer path in the catalyst under LCMM as illustrated in Figure 3b. In LCMM, the electrons may first pass from Cu atoms to lattice carbonate, which is then reduced to products. So, when electrons are injected into the Cu_2_(OH)_2_CO_3_ catalyst, Cu^2+^ will be temporarily reduced to Cu^+^ or Cu^0^. Then, after the electrons are further transferred to lattice carbonate, the Cu ions will return to Cu^2+^. Since the reaction kinetics are not fast enough, this intermediate state was detected. Moreover, the changes of lattice carbonate also agree with the electron transfer process, which is supported by XPS C 1s spectra in Figure 3c. To further reveal the real reaction, in situ Raman spectroscopy at different potentials was performed for these electrodes in a modified electrochemical cell. According to literatures, various Raman vibration of catalyst structure, such as OH─Cu─O^[^ ^20^ ^]^ at ≈169 and ≈206 cm^−2^ and surface intermediates,^[^ ^21^ ^]^ such as symmetric carbonate, ν_1_CO_3_^2−^ at 1065 cm^−2^, asymmetric carbonate, ν_2_CO_3_^2−^ at 840 cm^−2^, asymmetric CO_2_ activation species, ν_as_CO_2_^−^ at ≈1524 cm^−2^, CO intermediates, νCu─CO^[^ ^5a^ ^]^ at 298 cm^−2^ etc. were successfully detected (Figure S11, Supporting Information). However, the distribution of these vibration peaks for these electrodes is quite different.

As seen in Figure 3d, we observed that only the intensity of ν_1_CO_3_^2−^ for Cu_2_(OH)_2_CO_3_ electrode was greatly enhanced during the reaction compared with the Cu_2_O electrode, indicating that lattice carbonate mediated process accurately enhances the reactivity of a certain carbonate on the catalyst surface. Next, ν_s_CO_2_^−^ and ν_as_CO_2_^−^ were found in both electrodes, but deep reduction intermediates, νCu─CO, νCO, νC─O‐C, and νO─CH─O^[^ ^22^ ^]^ were only formed in Cu_2_(OH)_2_CO_3_ electrode, suggesting that ν_1_CO_3_^2−^ vibration enhancement in LCMM facilitates the activation and further reduction of CO_2_. At the same time, CO is widely accepted as a key intermediate for C_2+_ production. Thus, the easy generation of CO intermediates on LCMM catalyst surface may be beneficial to carbon–carbon coupling, thereby promoting the production of C_2+_ products, such as C_2_H_4_.

**Figure 4:**
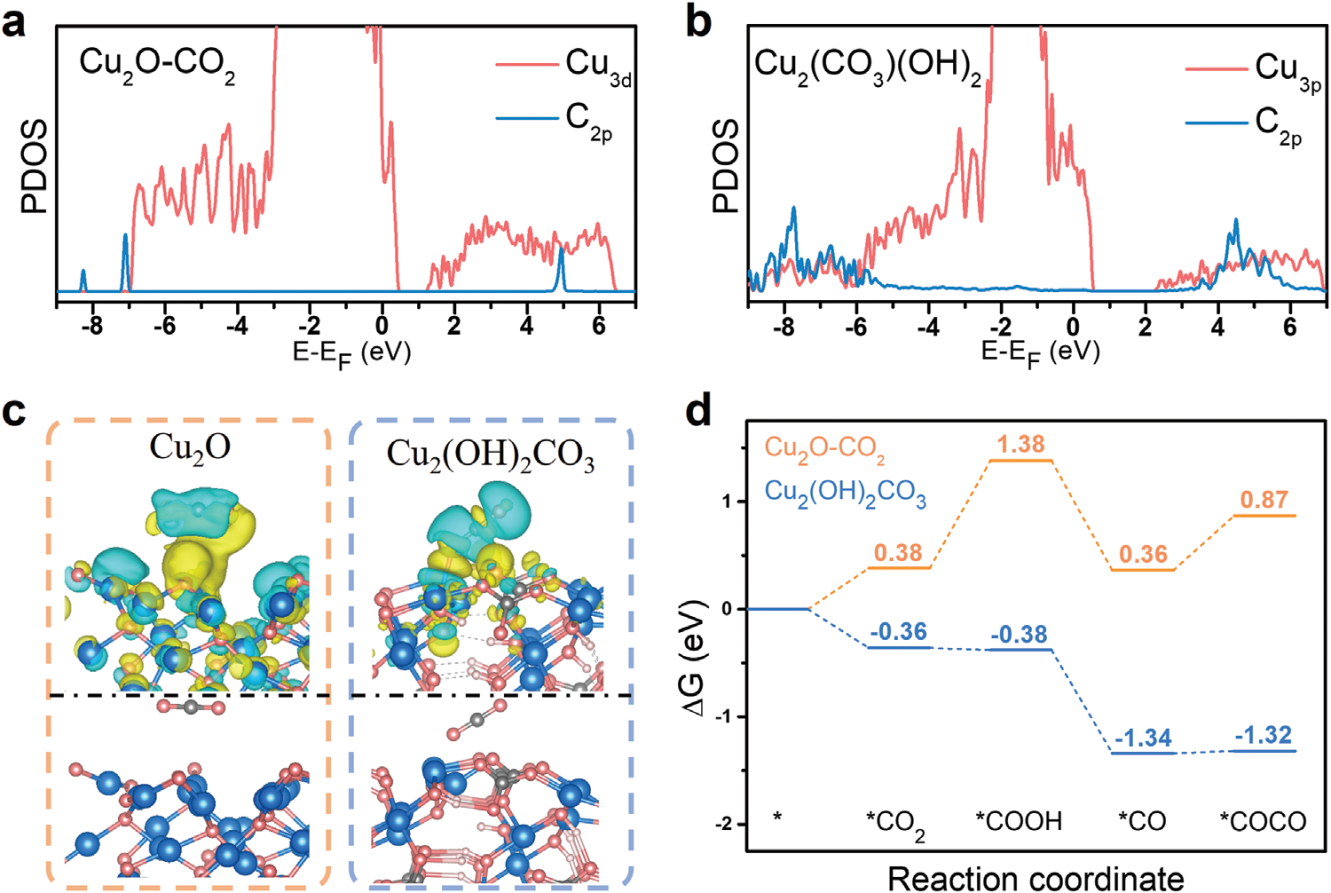
PDOS plot of Cu_3d_ and C_2p_ orbitals in a) Cu_2_O and b) Cu_2_(OH)_2_CO_3_, c) Charge density difference of Cu_2_O with adsorbed CO_2_ (left) and Cu_2_(OH)_2_CO_3_ with lattice carbonate (right), in which green and yellow regions indicate electron depletion and accumulation, respectively, d) Free energy diagrams of CO_2_ and lattice carbonate activation and coupling on Cu_2_O and Cu_2_(OH)_2_CO_3_, respectively.

To gain insight into the mechanism of LCMM on reaction kinetics, the projected density of states (PDOS) of Cu_3d_ and C_2p_ and charge density difference were calculated by DFT for Cu_2_(OH)_2_CO_3_ and Cu_2_O slabs (for details of our DFT calculations, see the computational section of SI). As seen in **Figure** **4**a,b, Cu_2_(OH)_2_CO_3_ with lattice carbonate has a higher overlap among the binding states between Cu_3d_ and C_2p_ than Cu_2_O with adsorbed CO_2_, resulting in a stronger binding energy of lattice CO_2_ on Cu_2_(OH)_2_CO_3_.^[^ ^23^ ^]^ This may reduce the energy barrier of CO_2_ transformation to CO_2_^−^ intermediates, thereby boosting CO_2_ activation and reduction. Then, their adsorption configurations and the corresponded charge density difference were illustrated in Figure 4c. It can be seen that CO_2_ in lattice carbonate is closer to the surface of the catalyst than Cu_2_O, which leads to easier electron transfer and less accumulation of electrons. To probe the effect of LCMM on the energy barrier of the reaction, the adsorption energies of CO_2_ on Cu_2_O slab and lattice carbonate on Cu_2_(OH)_2_CO_3_ slab were calculated, respectively, in Figure 4d and the adsorption configurations were shown in Figures S12 and S13 (Supporting Information)**_._** The energy barriers of lattice carbonate adsorption and activation on Cu_2_(OH)_2_CO_3_ is −0.36 and −0.02 eV, which are much negative than that of 0.38 and 1 eV for CO_2_ on Cu_2_O. This suggests that lattice carbonate reduction in LCMM is easier than the reduction of CO_2_ molecules on catalyst in AEM. Moreover, the energy barrier of the formation of *COCO on Cu_2_(OH)_2_CO_3_ was 0.02 eV, which was also much lower than that of 0.51 eV on Cu_2_O. These results prove that the enhanced electrocatalytic performance of CO_2_ reduction to C_2_H_4_ with LCMM in Cu_2_(OH)_2_CO_3_ is attributed to the easier electron transfer and the reduced energy barriers of CO_2_ activation and carbon–carbon coupling.

## 3 Conclusion

In conclusion, a lattice carbonate‐mediated mechanism, LCMM from Cu_2_(OH)_2_CO_3_ was revealed to promote the kinetics of electrocatalytic CO_2_ reduction to C_2_H_4_. Combined ^13^C IRMS and experiments, we showed that lattice carbonates acted as a mediator to link CO_2_ with the catalyst. By this route, Cu_2_(OH)_2_CO_3_ exhibited a FE_C2H4_ value of 60% and an intrinsic j_C2H4_ value of 29.0 mA cm^−2^ at a low overpotential of 0.97 V in H‐cell, which was ten‐fold and 15‐fold higher than that of Cu_2_O, respectively. In situ Raman spectrometry showed that LCMM catalyst could selectively enhance the reactivity of symmetric carbonates, thereby accelerating reaction kinetics. DFT calculations suggested that LCMM in Cu_2_(OH)_2_CO_3_ not only reduces the energy barrier of CO_2_ activation and C─C coupling, but also facilitates the electron transfer from Cu to CO_2_, thereby greatly boosting the electrocatalytic performance of CO_2_ reduction to C_2_H_4_. This work uncovers a novel lattice‐involved mechanism in electrocatalytic CO_2_RR for C_2_ production, which provides an effective route to fabricate efficient electrocatalysts.

## Conflict of Interest

The authors declare no conflict of interest.

## Supporting information

Supporting Information

## Data Availability

Research data are not shared.
