# Supplementary material for: Revealing the Lattice Carbonate Mediated Mechanism in Cu2(OH)2CO3 for Electrocatalytic Reduction of CO2 to C2H4
Source: Adv Sci (Weinh). 2024 Feb 4;11(14):2308949. doi: 10.1002/advs.202308949 (PMC11005744; doi:10.1002/advs.202308949)
Supplement: Supplementary file 1 — Supporting Information [file ADVS-11-2308949-s001.pdf]

ADVANCED SCIENCE
Open Access

Supporting Information

for *Adv. Sci.*, DOI 10.1002/advs.202308949

Revealing the Lattice Carbonate Mediated Mechanism in $Cu_2(OH)_2CO_3$ for Electrocatalytic Reduction of CO_2 to C_2H_4

Yugang Gao, Difei Xiao, Zeyan Wang, Zhaoke Zheng, Peng Wang, Hefeng Cheng, Yuanyuan Liu, Ying Dai and Baibiao Huang**

Supplementary Information

Revealing the Lattice Carbonate Mediated Mechanism in $Cu_2(OH)_2CO_3$ for Electrocatalytic Reduction of CO_2 to C_2H_4

Yugang Gao[a], Difei Xiao[a], Zeyan Wang[a,], Zhaoke Zheng[a], Peng Wang[a], Hefeng Cheng[a], Yuanyuan Liu[a], Ying Dai[b], Baibiao Huang[a,]**

[a] State Key Laboratory of Crystal Materials, Shandong University, Jinan 250100, China

[b] School of Physics, Shandong University, Jinan 250100, China

Abstract

Understanding CO_2 transformation mechanism on materials is essential for the design of efficient electrocatalysts for CO_2 reduction. In conventional adsorbate evolution mechanism (AEM), the catalysts encounter multiple high-energy barrier steps, especially CO_2 activation, limiting the activity and selectivity. Here, lattice carbonate from $Cu_2(OH)_2CO_3$ is revealed to be a mediator between CO_2 molecules and catalyst during CO_2 electroreduction by a ^{13}C isotope labelling method, which can bypass the high energy barrier of CO_2 activation and strongly enhance the performance. With the lattice carbonate mediated mechanism (LCMM), the $Cu_2(OH)_2CO_3$ electrode exhibited 10-fold faradaic efficiency and 15-fold current density for ethylene production than Cu_2O electrode with AEM at a low overpotential. Theoretical calculations and *in situ* Raman spectroscopy results show that symmetric vibration of carbonate is precisely enhanced on catalyst surface with LCMM, leading to faster electron transfer, lower energy barriers of CO_2 activation and carbon-carbon coupling. This work provides a route to develop efficient electrocatalysts for CO_2 reduction based on lattice mediated mechanism.

Corresponding authors:

Email: wangzeyan@sdu.edu.cn, bbhuang@sdu.edu.cn

Experimental section

Preparation of the $Cu_2(OH)_2CO_3$ nanoparticles on Cu foil. The pure Cu foil was washed with dilute hydrochloric acid to remove the oxide layer on the surface, and then stored in absolute ethanol as the substrate. The $Cu_2(OH)_2CO_3$ particles on Cu foil (3cm*2cm) was prepared in a two-electrode system using H_2O (30mL)/ethanol (30mL) of 0.2M Na_2CO_3 solution under the constant current of ~0.1A. When the bias on the electrode begins to rise rapidly, the experiments stopped and the $Cu_2(OH)_2CO_3$ particles were successfully prepared on Cu foil.

Preparation of the $Cu_2(OH)_2CO_3$ nanoparticles. $Cu_2(OH)_2CO_3$ nanoparticles were synthesized by a co-precipitation method according to previous reports[1,2]. In a typical synthesis, 100mL copper sulfate pentahydrate solution (1.0M) was added dropwise into 100 mL sodium carbonate solution (1.2M) at 60 °C. The suspension was then continuously stirred at 60 °C for 2 h. After that, the precipitate was collected by centrifugation and was washed by deionized water three times. Finally, the precipitate was dried under vacuum at 60 °C for 6 h to form as-prepared $Cu_2(OH)_2CO_3$ nanoparticles.

Preparation of the Cu_2O particles. Cu_2O particles were synthesized by wet chemical reduction method according to previous reports[3,4]. In a typical synthesis, 4g polyvinylpyrrolidone (PVP, MW 24000) was added into 100 mL $CuCl_2 \cdot 2H_2O$ aqueous solution (0.01M). Then, 10.0 mL NaOH aqueous solution (2.0 M) was added dropwise into the above solution. After stirring for 30 min, 10.0 mL ascorbic acid solution (0.60 M) was added dropwise into the dark brown solution. The mixture was aged for 3 h and the solution gradually transferred into turbid red. All of the procedure was carried out

under constant stirring and heated in a water bath at 55 °C. The resulting precipitate was collected by centrifugation and decanting, followed by washing with distilled water 3 times, absolute ethanol 3 times and deionized water 3 times again. Finally, the precipitate was dried under vacuum at 60 °C for 6 h.

Preparation of $Cu_2(OH)_2CO_3$ and Cu_2O electrodes. Typically, a suspension of nanoparticles was prepared by adding 10mg catalyst ($Cu_2(OH)_2CO_3$, Cu_2O nanoparticles) into the solution-base of 200 μL of isopropanol, 20 μL of H_2O and 40 μL of 5% nafion solution. And then the suspension was ultrasonicated for 3h to make it homogeneously dispersed. We deposited 10 μL of the sample mixtures on the GCE (diameter, 5mm) to form the sample electrodes.

Electrochemical experiments. The linear sweeping voltammetry (LSV) measurements were carried out with an Ag/AgCl reference electrode (with saturated KCl as the filling solution), a platinum electrode as the counter electrode and the as-prepared samples as the working electrode. The product analysis was carried out in a two-compartment electrochemical cell with an anion exchange membrane separating the working and counter electrodes. The potentiostatic measurements were performed using a three-electrode system to determine the value of the consumed coulomb, and the amounts of the gases produced were measured by the GC and GCMS instruments. The electrolyte was potassium bicarbonate saturated with CO_2 by bubbling high-purity CO_2 gas, before each experiment, at a flow rate of 50 ml min^{-1} for 1 h to remove all oxygen from the electrolyte. The working electrode was tested 20 times before the plot is recorded at a scan rate of 50 mV s^{-1}. All potentials were transformed to the reversible

hydrogen electrode (RHE) reference by using the calibrated relationship, E_{RHE} = $E_{Ag/AgCl}$ +0.657V.

Characterization. Crystal structures of the as-obtained products were characterized by XRD measurements with a Bruker AXS D8 diffractometer using Cu Kα radiation. Fourier transform infrared (FTIR) spectra were obtained on a Bruker ALPHA-T spectrometer using KBr pellets. Raman spectra were recorded on a microscopic confocal Raman spectrometer (Horiba JobinYvon, LabRAM HR) with an excitation of 613 nm laser light. Morphologies and microstructures of the products were characterized by transmission electron microscopy (TEM) using a Philips Tecnai 20U-Twin microscope at an acceleration voltage of 200 kV. (JEOL JEM-2100F). X-ray photoelectron spectroscopy (XPS) measurement was performed using a Thermo Fisher Scientific Escalab 250 spectrometer with monochromatized Al Kα excitation, and C1s (284.6 eV) was used to calibrate the peak positions of various elements. All electrochemical experiments were carried out using the electrochemical workstation CHI660E. The gas products from the compartment were examined with a gas chromatograph (GC) equipped with a TDX-01 column with a flame ionization detector (FID) and a H_2-detection GC (ShiweipxGC-7806) with a thermal conductivity detector (TCD). Gas chromatograph-mass spectrometer (GCMS) was used to determine the concentration of liquid products with a Max capillary column.

The amount of ^{13}C in electrocatalyst is tested by stable isotope ratio mass spectrometry using a Elementar Isoprime 100. We adopted stable isotope ratio mass spectrometry to detect the amount of ^{13}C in $Cu_2(OH)_2CO_3$ before and after reaction. Since IRMS can

only test powder samples, we need to scrape the $Cu_2(OH)_2CO_3$ powder from the electrode after reaction for detection. Finally, we can obtain ^{13}C results from IRMS, namely ^{13}C intensity from MS detection and δ ^{13}C values obtained through calculations according to the equation, δ (‰) = (R_{sq}/R_{st}-1)×1000, where R is defined as the ratio of the abundance of ^{13}C isotope atoms to the abundance of ^{12}C isotope atoms, R_{sq} and R_{st} are refer to isotope ratio of the sample and the standard material, respectively.

The faradaic efficiency (FE) was calculated by the following equation

$$FE_{C2H4} = \frac{\alpha nF}{Q} = \frac{12nF}{It}$$

Where α is the number of the electrons transferred for C_2H_4, F is the Faraday constant, Q is the charge, *I* is the current, t is the running time and n is the total amount of C_2H_4 (in moles).

RHE calibration

The RHE calibration is performed under the practical condition according to the previous reports (**Figure S1**) [5,6]. The calibration was carried out in the high purity hydrogen and CO_2 saturated $KHCO_3$ electrolyte with a Pt wire as the working electrode. Cycle Voltammetry was run at a scan rate of 1 mV s^{-1}. The average of the two potentials at which the current crossed zero was taken to be the thermodynamic potential for the hydrogen electrode reactions. So, in CO_2 saturated 0.5M $KHCO_3$, E (RHE) = E (Ag/AgCl) + 0.657 V.

CO_2 saturated 0.5M $KHCO_3$

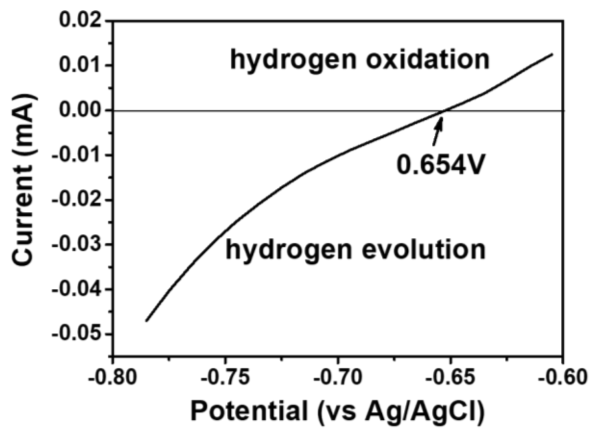

Figure S1. Calibration of Ag/AgCl reference electrode relative to the RHE.

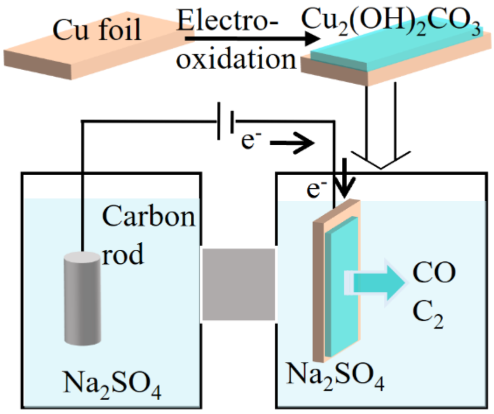

Figure S2. The growth of $Cu_2(OH)_2CO_3$ NPs on Cu foil by an eletro-oxidation method and the following electrocatalytic tests.

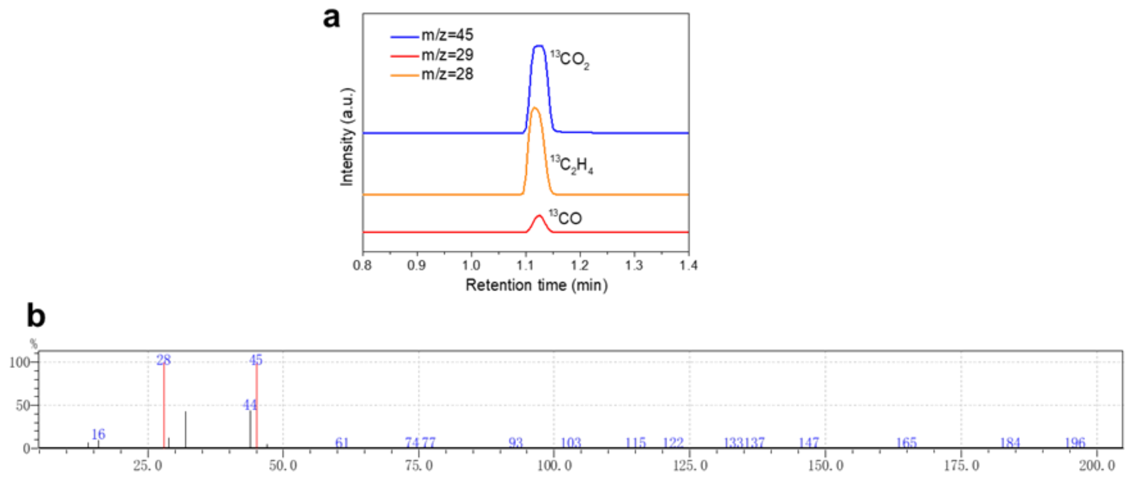

Figure S3. (a) Extracted ion chromatogram at different mass to charge ratios and (b) Mass chromatograph of products.

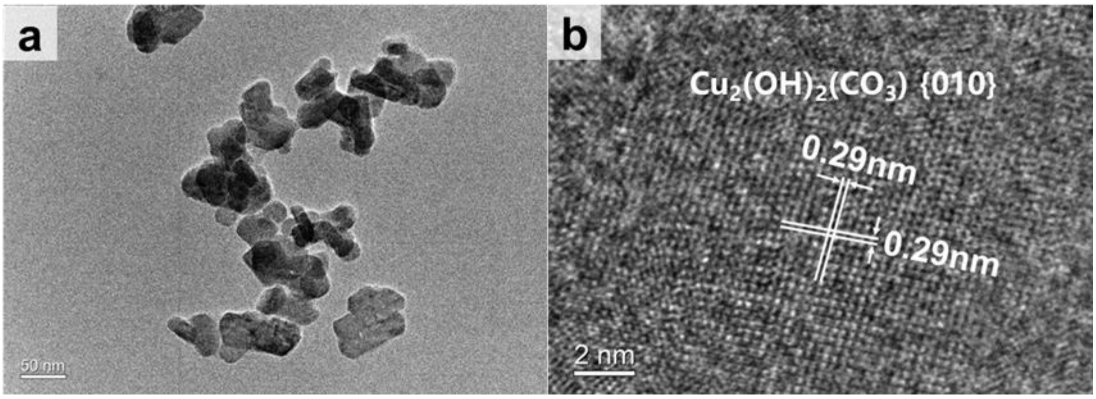

Figure S4. (a) TEM and (b) High resolution TEM images with lattice fringes for $Cu_2(OH)_2CO_3$ electrode before reaction.

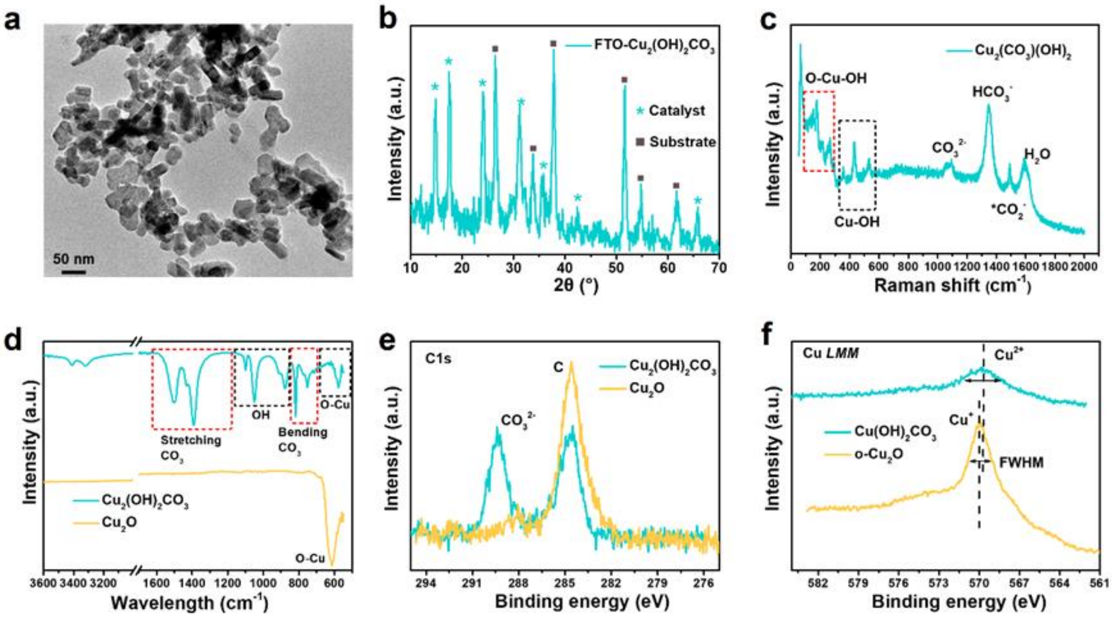

Figure S5. (a) TEM image, (b) XRD pattern, (c) Raman spectra for $Cu_2(OH)_2CO_3$ NPs, (d) FT-IR spectra, (e) High resolution C1s XPS spectra, (f) High resolution Cu *LMM* Auger spectra for $Cu_2(OH)_2CO_3$ and Cu_2O NPs.

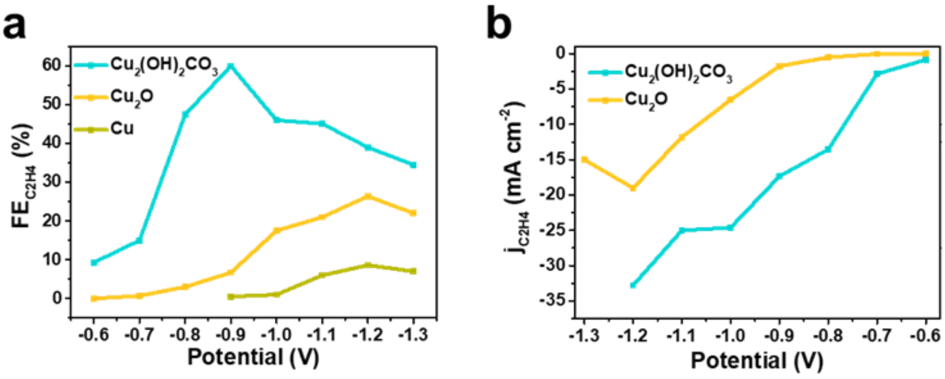

Figure S6. (a) The FE_{C2H4} values of $Cu_2(OH)_2CO_3$, Cu_2O and Cu electrodes at various potentials, (b) the j_{C2H4} values at various potentials relative to geometric surface areas.

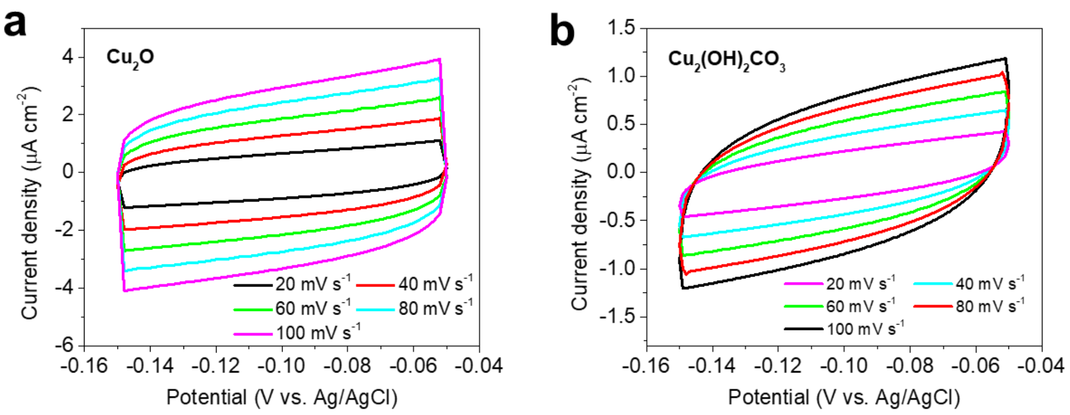

Sample	R_f	ECSA(cm^{-2})
Cu_2O	12.0	2.5
$Cu_2(OH)_2CO_3$	2.9	0.6

Figure S7. Cyclic voltammetry measurements in Ar-saturated 0.1M KOH for (a) Cu_2O electrode and (b) $Cu_2(OH)_2CO_3$ electrode at various scan rates.

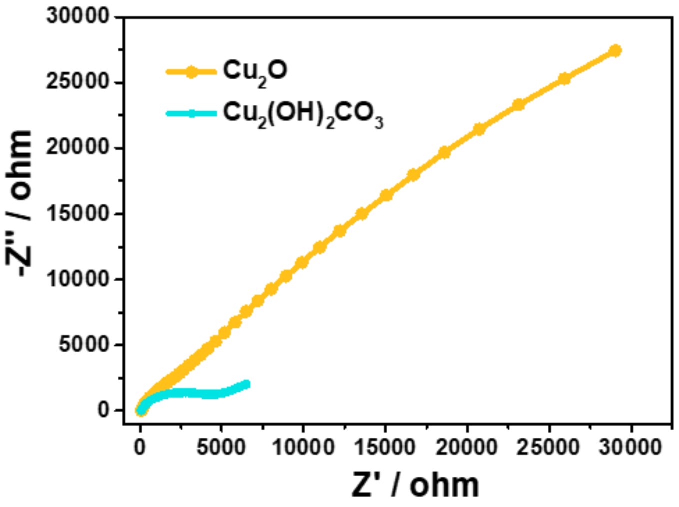

Figure S8. EIS spectra of $Cu_2(OH)_2CO_3$ and Cu_2O electrodes recorded at -0.6V.

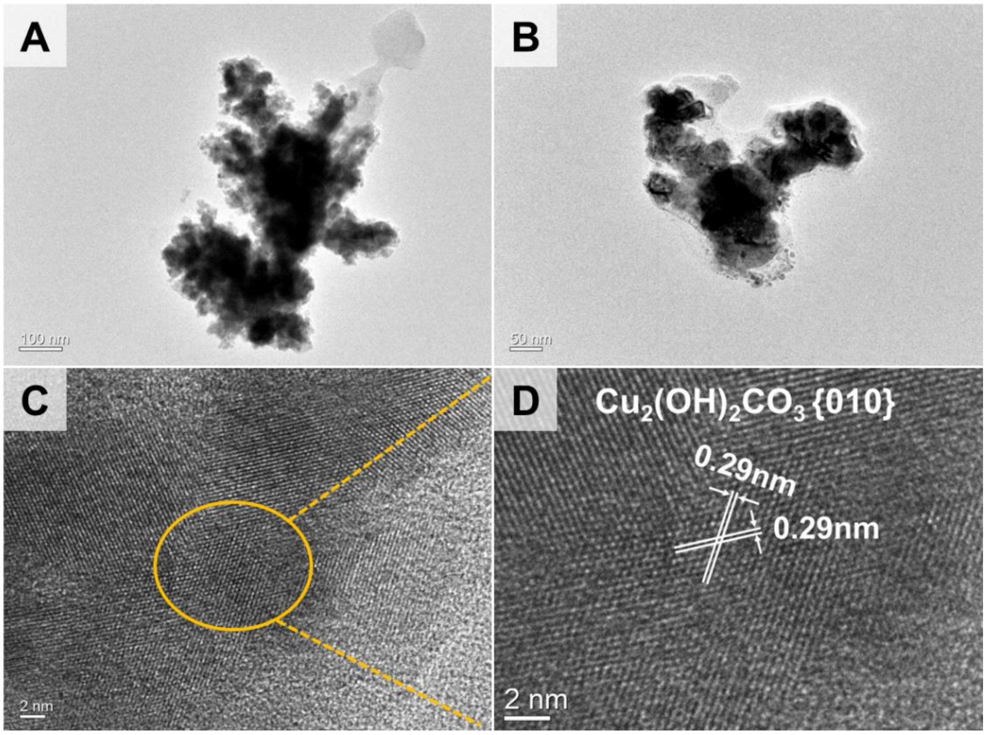

Figure S9. (a, b) TEM and (c, d) High resolution TEM images for $Cu_2(OH)_2CO_3$ electrode after reaction.

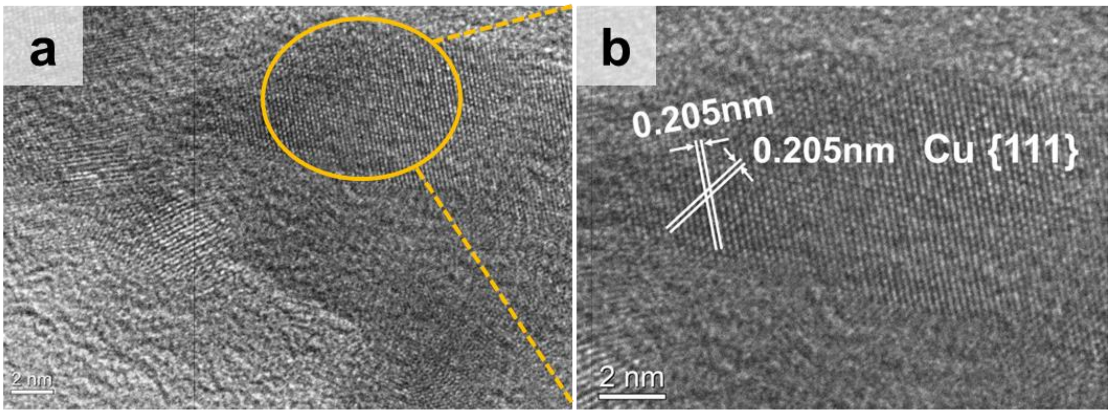

Figure S10. (a) TEM and (b) High resolution TEM images for $Cu_2(OH)_2CO_3$ electrode after reaction.

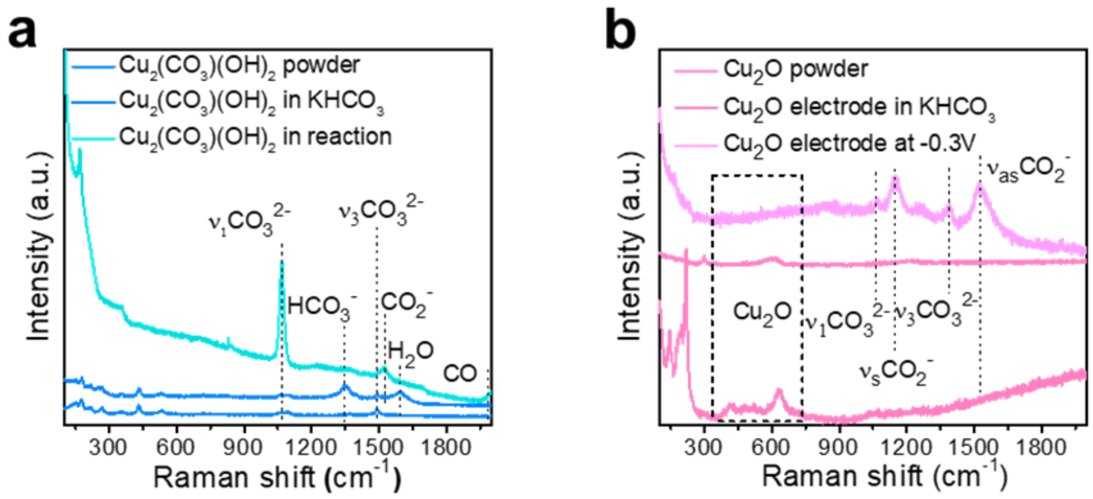

Figure S11. Raman spectra of (a) $Cu_2(OH)_2CO_3$ and (b) Cu_2O in the powder state, electrode unreacted state, and reaction state.

Table S1. Comparison of Electrocatalytic performance of CO_2RR to C_2H_4 for various catalysts in H-cells.

Sample	Electrolyte	J_{C2H4} (mA/cm^2)	FE_{C2H4} (%)	Overpotential η_{C2H4} (V)	Ref.
$Cu_2(CO_3)(OH)_2$	0.5M $KHCO_3$	30.2	60	0.97	This work
CuO-CTAB	0.5M $KHCO_3$	7.5	58.3	1.15	7
Electrodeposited Cu_2O	0.5M $KHCO_3$	2.5	26	1.19	8
Malachite nanorods	0.1M $KHCO_3$	0.24	3.1	1.05	9
Electro-redeposited Cu	0.1M $KHCO_3$	22.2	38	1.27	10
Plasma Cu	0.1M $KHCO_3$	7.2	60	0.97	11
Cu/Cu_3N	0.1M $KHCO_3$	10.4	43	1.07	12
Cycled Cu nanocubes	0.25M $KHCO_3$	22.4	32	1.02	13
Fragmented Cu_2O	0.1M $KHCO_3$	10.0	57	1.17	14
Cu nanowires	0.1M $KHCO_3$	17.3	77	1.07	15
Branched CuO NPs	0.1M $KHCO_3$	17.0	70	1.12	16

Computational section

All calculations were performed by using the Vienna Ab Initio Simulation Package (VASP)[17,18] with the projector augmented wave (PAW) method.[18] We employed the generalized gradient approximation (GGA) of the Perdew−Burke−Ernzerhof (PBE) exchange-correlation functional[19] and a cutoff energy of 500 eV. The van der Waals (vdW) interactions were taken into account by the empirical correction scheme of Grimme (DFT+D2).[20] A Monkhorst-Pack 5 × 5 × 1 k-point grid was adopted for all the calculations. The convergence criterion for the residual force and energy was set to 0.01 eV/Å and 10^{-4} eV with a vacuum space larger than 20 Å in the z direction to avoid interactions between periodic units during the structure relaxation.

The computational hydrogen electrode (CHE)[21] model was used to compute the Gibbs reaction free energy change (ΔG) of each elementary step for NOER. The ΔG of every step is obtained by

$$\Delta G = \Delta E + \Delta E_{\mathrm{ZPE}} - T\Delta S$$

where ΔE is the enthalpy difference from DFT computations, ΔE_{ZPE} is the change in zero point energy, T and ΔS is the temperature (298.15 K) and the entropy changes, which can be calculated from the vibrational frequencies.

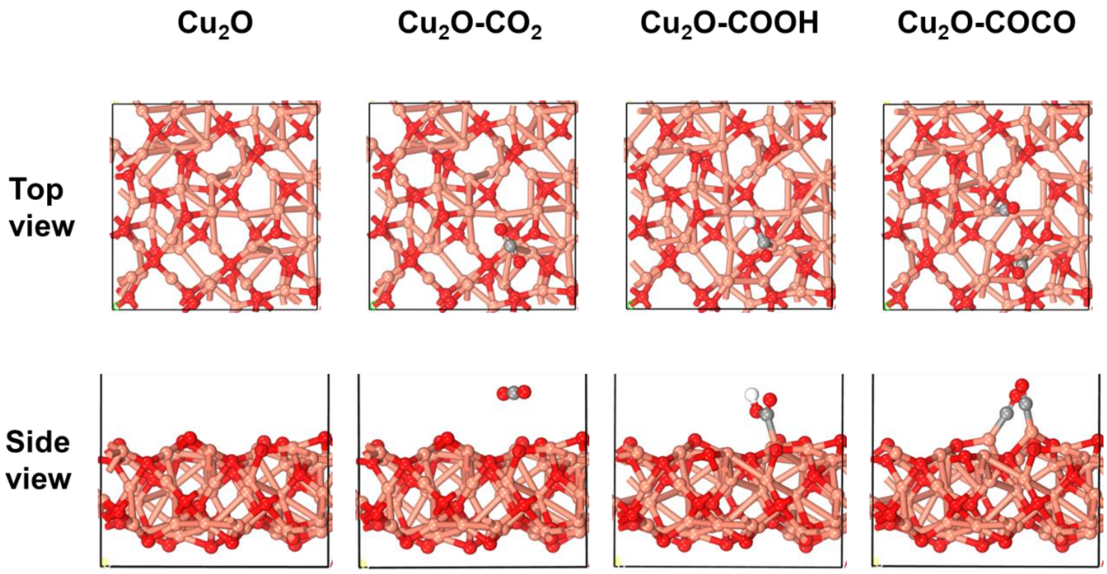

Figure S12. Top views and side views of Cu_2O slabs with CO_2, COOH, CO-CO adsorbed on the surface.

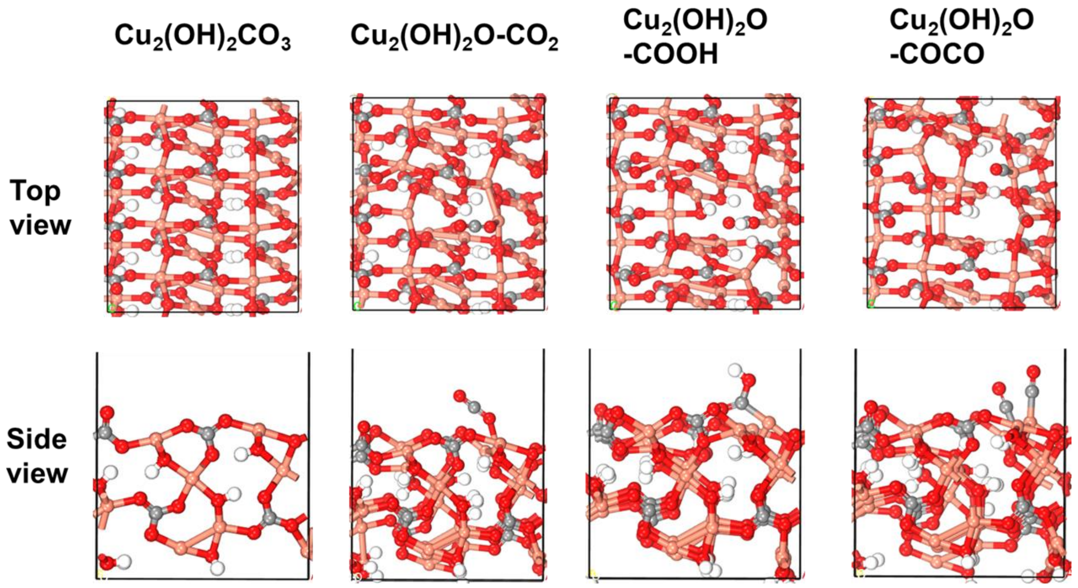

Figure S13. Top views and side views of $Cu_2(OH)_2CO_3$ slabs with lattice CO_2, active CO_2, COOH, CO-CO adsorbed on the surface.

References

[1] M. Behrens, F. Girgsdies, A. Trunschke, R. Schlögl, *Eur. J. Inorg. Chem.* **2009**, *2009*, 1347-1357.

[2] B. Bems, M. Schur, A. Dassenoy, H. Junkes, D. Herein, R. Schlogl, *Chem. Eur. J* **2003**, *9*, 2039-2052.

[3] D.-F. Zhang, H. Zhang, L. Guo, K. Zheng, X.-D. Han, Z. Zhang, *Journal of Materials Chemistry* **2009**, 19, 5220.

[4] J. Lin, W. Hao, Y. Shang, X. Wang, D. Qiu, G. Ma, C. Chen, S. Li, L. Guo, *Small* **2018**, 14, 1703274.

[5] Y. Liang, Y. Li, H. Wang, J. Zhou, J. Wang, T. Regier and H. Dai, Nat Mater, **2011**, 10, 780-786.

[6] Y. Li, W. Zhou, H. Wang, L. Xie, Y. Liang, F. Wei, J. C. Idrobo, S. J. Pennycook and H. Dai, Nat Nanotechnol, **2012**, 7, 394-400.

[7] D. Zhao, S. Zhang, T. Guo, Q. Zhao, J. Du, J. Li, *ChemCatChem* 2023, 15, e202201413.

[8] D. Kim, S. Lee, J. D. Ocon, B. Jeong, J. K. Lee, J. Lee, *Phys. Chem. Chem. Phys.* **2015**, *17*, 824-830.

[9] M. Spodaryk, K. Zhao, J. Zhang, E. Oveisi, A. Züttel, *Electrochimica Acta* **2019**, *297*, 55-60.

[10] P. De Luna, R. Quintero-Bermudez, C.-T. Dinh, M. B. Ross, O. S. Bushuyev, P.

Todorović, T. Regier, S. O. Kelley, P. Yang, E. H. Sargent, *Nat. Catal.* **2018**, *1*, 103-110.

[11] H. Mistry, A. S. Varela, C. S. Bonifacio, I. Zegkinoglou, I. Sinev, Y. W. Choi, K. Kisslinger, E. A. Stach, J. C. Yang, P. Strasser, B. R. Cuenya, *Nat. Commun.* **2016**, *7*, 12123.

[12] Z. Q. Liang, T. T. Zhuang, A. Seifitokaldani, J. Li, C. W. Huang, C. S. Tan, Y. Li, P. De Luna, C. T. Dinh, Y. Hu, Q. Xiao, P. L. Hsieh, Y. Wang, F. Li, R. Quintero-Bermudez, Y. Zhou, P. Chen, Y. Pang, S. C. Lo, L. J. Chen, H. Tan, Z. Xu, S. Zhao, D. Sinton, E. H. Sargent, *Nat. Commun.* **2018**, *9*, 3828.

[13] K. Jiang, R. B. Sandberg, A. J. Akey, X. Liu, D. C. Bell, J. K. Nørskov, K. Chan, H. Wang, *Nat. Catal.* **2018**, *1*, 111-119.

[14] H. Jung, S. Y. Lee, C. W. Lee, M. K. Cho, D. H. Won, C. Kim, H. S. Oh, B. K. Min, Y. J. Hwang, *J. Am. Chem. Soc.* **2019**, *141*, 4624-4633.

[15] C. Choi, S. Kwon, T. Cheng, M. Xu, P. Tieu, C. Lee, J. Cai, H. M. Lee, X. Pan, X. Duan, W. A. Goddard, Y. Huang, *Nat. Catal.* **2020**, *3*, 804-812.

[16] J. Kim, W. Choi, J. W. Park, C. Kim, M. Kim, H. Song, *J. Am. Chem. Soc.* **2019**, *141*, 6986-6994.

[17] G. Kresse, J. Furthmüller, J. *Phys. Rev. B: Condens. Matter Mater. Phys*. **1996**, *54*, 11169.

[18] G. Kresse, D.Joubert, *Phys. Rev. B: Condens. Matter Mater. Phys*. **1999**, *59*, 1758−1775.

[19] J. P. Perdew, K. Burke, Ernzerhof, M. *Phys. Rev. Lett.* **1996**, *77*, 3865.

[20] S. Grimme, *J. Comput. Chem.*, **2006**, *27*, 1787–1799.

[21] J. K. Nørskov, J. Rossmeisl, A. Logadottir, L. Lindqvist, J. R. Kitchin, T. Bligaard, H. J´onsson, *J. Phys. Chem. B* **2004**, *108*, 17886–17892.
